# CODEHOP-Mediated PCR Improves HIV-1 Genotyping and Detection of Variants by MinION Sequencing

**DOI:** 10.1128/Spectrum.01432-21

**Published:** 2021-10-20

**Authors:** Horeyah Sarkhouh, Wassim Chehadeh

**Affiliations:** a Department of Microbiology, Faculty of Medicine, Kuwait University, Jabriya, Kuwait; Johns Hopkins Hospital

**Keywords:** HIV-1 genotyping, drug resistance, CODEHOP, MinION sequencing, variants

## Abstract

HIV-1 is genetically heterogeneous, having different subtypes and circulating recombinant forms (CRFs). HIV-1 genotyping is used to determine drug resistance profiles and is based on the use of a mixture of consensus and degenerate primers targeting the *pol* gene. However, the use of this type of primers is associated with either PCR bias or PCR failure. Consensus-degenerate hybrid oligonucleotide primers (CODEHOPs) can detect and identify unknown and distantly related gene sequences by PCR. CODEHOPs designed using different HIV-1 subtypes and CRFs were evaluated for HIV-1 genotyping by Sanger and MinION sequencing. A total of 321 plasma samples were used for the validation of CODEHOP-mediated HIV-1 genotyping. CODEHOP-mediated PCR showed 100% sensitivity and specificity, with limits of detection and genotyping below 200 copies/ml. The head-to-head evaluation of CODEHOP-mediated PCR and standard PCR showed 97 to 98% and 82 to 84% PCR success rates, respectively. There was 100% agreement between the CODEHOP and the reference method in the drug resistance profiles determined by Sanger-based sequencing. Using MinION sequencing, the CODEHOP-mediated PCR scheme resulted in better depth of genome coverage and detection of more drug resistance variants in the protease and reverse transcriptase genes than the standard amplification scheme. The overall prevalences of drug resistance mutations were 17.1% in treatment-experienced patients and 1.2% in treatment-naive patients. They were mainly associated with resistance to reverse transcriptase inhibitors and were linked to virological failure and the patient’s treatment history. Findings from this study suggest that the performance of HIV-1 genotyping is improved by using CODEHOP-mediated PCR.

**IMPORTANCE** HIV-1 drug resistance is the main cause of treatment failure. Regular surveillance of resistance-associated mutations in HIV-1 genomes is essential for the optimal management of HIV-1 infections. Due to HIV-1’s genetic diversity, different HIV-1 genotypes are circulating worldwide. Standard primers used in the amplification of HIV-1 RNA have not been designed to cover all HIV-1 genotypes and are the main cause of amplification and drug resistance test failure. In this study, new sets of PCR primers targeting the protease, reverse transcriptase, and integrase genes were designed using the CODEHOP approach. They were compared to primers recommended in part by WHO for drug resistance testing using in-house PCR. Unsuccessful HIV-1 RNA amplification was less likely to occur with CODEHOP primers, leading to fewer test failures and lower cost. Furthermore, CODEHOP primers were more effective than standard primers for the detection of minority resistant variants by MinION sequencing.

## INTRODUCTION

HIV-1 group M is predominant worldwide and is currently divided into subtypes A1, A2, A3, A4, B, C, D, F1, F2, G, H, J, and K, with genetic variation ranging between 25% and 35%. The genetic variation within an HIV-1 subtype can be between 15% and 20% (1). In addition to HIV-1 subtypes, there are circulating recombinant forms (CRFs) and unique recombinant forms (URFs), which are the result of infection of the same cell with two or several subtypes (2). There are currently more than 100 HIV-1 CRFs described in the Los Alamos National Laboratory HIV-1 Sequence Database (3).

HIV-1 in any clinical sample is genetically heterogeneous, and in heavily treated patients, about 1% of positions show evidence of a nucleotide mixture (4). Most mutations in HIV-1 genomes result from the absence of proofreading activity in the reverse transcriptase (5) and from the actions of mutagenic enzymes like APOBEC3G (6). There are two types of mutations that are associated with drug resistance, the primary mutations that lead directly to drug resistance and generally to a decrease in viral fitness, and the secondary mutations that result from continued drug selective pressure and are generally associated with an improved viral fitness (7). Resistance-associated mutations are usually detected by Sanger-based sequencing of the *pol* gene following the isolation of HIV RNA from plasma. Next-generation sequencing (NGS) technologies have offered the opportunity to detect population diversity, including minor variants (8–10).

Pools of consensus and degenerate primers with nucleotide sequence differences covering all possible nucleotide variations in the HIV-1 sequence have been used to amplify the HIV-1 *pol* gene (4). The main drawback of using degenerate and consensus primers in PCR is the amplification of several copies of DNA from a small copy number of an HIV genome, masking the presence of different variants potentially present in the original population and creating sequence resampling (11). Furthermore, nucleotide polymorphisms and intersubtype genetic variability may disrupt primer annealing and complicate HIV-1 genotyping, especially when HIV-1 sequences are more distantly related or are in low copy numbers (12, 13).

Consensus-degenerate hybrid oligonucleotide primers (CODEHOPs) have been developed previously to detect and identify unknown and distantly related gene sequences by PCR (14–16). A CODEHOP consists of two regions, a short 3′ degenerate core region that corresponds to all possible codons specifying 3 or 4 highly conserved amino acids and a long 5′ consensus clamp region that contains the consensus sequences flanking the target motif. This increases the efficiency of PCR, allowing the amplification of distantly related sequences in complex viral populations (17). CODEHOP-mediated PCR was reported to be more reliable and sensitive than standard diagnostic techniques in the detection and typing of enteroviruses and human papillomaviruses in clinical samples (18, 19). However, it has not been investigated in the detection and genotyping of HIV-1 drug-resistant variants. In this study, CODEHOPs were designed as universal primers to amplify different HIV-1 subtypes and were compared to the standard primers in HIV-1 genotyping by Sanger and MinION sequencing.

## RESULTS

### Evaluation of the CODEHOP-mediated PCR.

**FIG 1:**
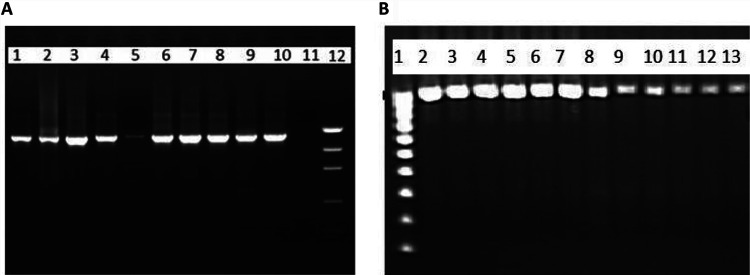
CODEHOP-mediated PCR amplification of the *pol* gene from different HIV-1 CRFs. (A) Nested RT-PCR amplification of protease and reverse transcriptase genes. Lane 1, CRF35_AD; lane 2, CRF07_BC; lane 3, CRF08_BC; lane 4, CRF63_02A; lane 5, CRF67_01B; lane 6, CRF16_A2D; lane 7, CRF10_CD; lane 8, CRF02_AG; lane 9, CRF50_A1D; lane 10, CRF43_02G; lane 11, negative control; lane 12, high-DNA mass ladder. (B) Seminested RT-PCR amplification of integrase gene. Lane 1, low-DNA mass ladder, lane 2, CRF35_AD; lane 3, CRF07_BC; lane 4, CRF08_BC; lane 5, CRF63_02A; lane 6, CRF67_01B; lane 7, CRF16_A2D; lane 8, CRF10_CD; lane 9, CRF02_AG; lane 10, CRF50_A1D; lane 11, CRF43_02G; lane 12, CRF32_06A1; lane 13, CRF25_cpx.

A total of 35 CODEHOPs were designed using the j-CODEHOP tool (Table S1 in the supplemental material). When the primers were pooled, multiplex PCR resulted in no amplicons or multiple amplicons with different sizes depending on the HIV-1 subtype. Different combinations of forward and reverse primers in nested and seminested reverse transcription-PCR (RT-PCR) were then tested to amplify the *pol* gene of 18 different HIV-1 subtypes. CODEHOP pairs that produced no bands or nonspecific bands in one or more HIV-1 subtypes were excluded from further analysis. CODEHOP pairs that produced bands at expected sizes are listed in Table 1. The amplification of the protease region together with the reverse transcriptase region was successful for all 18 HIV-1 subtypes using one-step nested RT-PCR, generating an ∼1,700-bp band, whereas a one-step seminested RT-PCR with three different primers was the best approach to amplify the entire integrase region, generating an ∼1,050-bp band (Fig. 1). A single CODEHOP-mediated PCR amplicon spanning the protease, reverse transcriptase, and integrase genes could not be obtained for all HIV-1 subtypes tested. Direct sequencing of all PCR amplicons by the Sanger method confirmed the correct annealing of CODEHOPs and the amplification of the *pol* gene (data not shown).

**TABLE 1:** HIV-1 primers designed by using the j-CODEHOP tool

Primer	Target^*a*^	Sequence (5′→3′)	Polarity	Position^*b*^	Usage^*c*^
HPF1	PR/RT	CCATAAAGCAAGRGTKTTRG	Forward	1860–1879	RT-PCR
HPR1	PR/RT	CCATGTTTCTTTYGKKATRGG	Reverse	3743–3723	RT-PCR
HPF2	PR/RT	TAGGAAAAARGGYTGTTGGA	Forward	2013–2032	nPCR
HPR2	PR/RT	AAATTTAGGRRTYTTYCCCCA	Reverse	3716–3696	nPCR
HRTSF	PR/RT	GATCAGATACYYRTAGAVAT	Forward	2430–2449	Sequencing
HPSR	PR/RT	TACTAATTTTCTCCAYTTIGT	Reverse	2774–2754	Sequencing
HINF1	IN	CCAGATAAGAGTGARKCAGA	Forward	4077–4096	RT-PCR
HINR	IN	GGGATGTGTACTTCTGARCTT	Reverse	5213–5193	RT-PCR, snPCR, sequencing
HINF2	IN	CCAGCACAYAARGGRATTGG	Forward	4158–4177	snPCR, sequencing

a PR, protease; RT, reverse transcriptase; IN, integrase.

b Primer position according to the HIV-1 HXB2 reference strain sequence (accession no. K03455).

c nPCR, nested PCR; snPCR: seminested PCR.

### Validation of the CODEHOP-mediated PCR in HIV-1 genotyping.

The primers designed by the j-CODEHOP tool showed no cross-reactivity to a panel of viruses and samples from patients with active blood-borne virus infections (Fig. S1). CODEHOP-mediated PCR for HIV-1 genotyping was validated using 100 positive and 100 negative residual samples (Table 2). HIV-1 subtype assignment and viral load by individual sample are shown in Table S2. There was 100% agreement with the reference technique, yielding 100% sensitivity and 100% specificity. Table 3 summarizes the distribution of HIV-1 subtypes among tested samples. The limits of detection and successful genotyping with Sanger sequencing at 95% probability were ∼137 copies/ml (95% confidence interval [CI], 123.3 to 231.1) for the protease/reverse transcriptase genes and ∼117 copies/ml (95% CI, 83.1 to 246.5) for the integrase gene (Table 4). The head-to-head evaluation of CODEHOP-mediated PCR and standard PCR for genotyping of 121 samples showed 100% agreement. However, the failure rates of gene amplification of protease/reverse transcriptase and integrase genes using one-step RT-PCR in the first PCR run in the CODEHOP protocol were 2% and 3%, respectively, compared to 16% and 18% in the standard protocol (Table 4). The risk of failure to amplify the protease/reverse transcriptase gene using one-step RT-PCR followed by a nested PCR was 7.3 times higher with the standard protocol than with the CODEHOP protocol (95% CI, 2.1 to 39.5; *P < *0.001). Similar results were obtained for the integrase gene, with a higher PCR failure rate using the standard protocol than using the CODEHOP protocol (odds ratio, 6.5; 95% CI, 2.1 to 26.6; *P < *0.001). The highest PCR failure rates were mostly observed with HIV-1 subtypes C and CRF01_AE (Table 4).

**TABLE 2:** Validation of CODEHOP-mediated PCR in HIV-1 genotyping

HIV-1 subtype	Total no. of samples	No. of samples with indicated result	
		True positive	False negative
A	3	3	0
B	15	15	0
C	19	19	0
G	1	1	0
CRF01_AE	17	17	0
CRF02_AG	24	24	0
CRF06_cpx	6	6	0
CRF07_BC	5	5	0
CRF08_BC	1	1	0
CRF10_CD	1	1	0
CRF16_A2D	2	2	0
CRF25_cpx	1	1	0
CRF35_AD	1	1	0
CRF43_02G	2	2	0
CRF50_A1D	1	1	0
CRF63_02A1	1	1	0

Total	100	100	0

**TABLE 3:** Sensitivity of CODEHOP-mediated RT-PCR in HIV-1 genotyping

Viral load (copies/ml)	No. of replicates	Result using^*a*^:					
		Protease/reverse transcriptase			Integrase		
		No. Positive	% Positive	Probit value	No. Positive	% Positive	Probit value
1,000	12	12	100	NA	12	100	NA
500	12	12	100	NA	12	100	NA
250	12	12	100	NA	12	100	NA
125	12	10	83.3	5.94	12	100	NA
100	12	3	25	4.22	10	83.3	6.21
50	12	1	8.3	−1.1	3	25	4.28
10	12	0	0	NA	0	0	NA

a NA, not applicable.

**TABLE 4:** Head-to-head evaluation of CODEHOP-mediated RT-PCR and standard protocol

HIV-1 subtype	No. of samples	No. of samples positive using the indicate protocol^*a*^							
		CODEHOP				Standard			
		One-step RT-PCR		Two-step RT-PCR		One-step RT-PCR		Two-step RT-PCR	
		PR/RT	IN	PR/RT	IN	PR/RT	IN	PR/RT	IN
B	24	24	24			22	22	2	2
C	25	23	23	2	2	19	18	6	7
CRF01_AE	26	25	25	1	1	20	19	6	7
CRF02_AG	33	33	32		1	30	29	3	4
CRF06_cpx	12	12	12			10	10	2	2
CRF43_02G	1	1	1			1	1		

Total	121	118	117	3	4	102	99	19	22

a PR, protease; RT, reverse transcriptase; IN, integrase.

The total number of primers used to perform HIV-1 drug resistance testing using the standard protocol was 15, whereas only 9 primers were needed for the CODEHOP protocol. Amplification of protease and reverse transcriptase genes with the standard protocol was carried out using two separate nested PCRs, whereas one nested PCR was used in the CODEHOP protocol. The cost of the CODEHOP protocol, including the extraction, amplification, purification, and sequencing steps, was estimated at $89.60 per sample, whereas the standard amplification protocol cost $163.80 per sample. When the one-step RT-PCR failed and the two-step RT-PCR was performed, the cost of the CODEHOP protocol was increased to $95.20 and that of the standard protocol to $172.20.

Drug resistance mutations (DRMs) were detected in 4 (11.4%) treatment-experienced patients and 1 (1.2%) treatment-naive patient by the Sanger sequencing method, with no difference observed between the CODEHOP and the standard method (Table 5). Two sequences (from patients 2 and 3) had mutations associated with resistance to nucleoside/nucleotide-analogue reverse transcriptase inhibitors (NRTIs), one sequence (from patient 5) had a mutation associated with nonnucleoside reverse transcriptase inhibitors (NNRTIs), one sequence (from patient 1) had two mutations associated with resistance to NRTIs and NNRTIs, and one sequence (from patient 4) had one mutation associated with resistance to protease inhibitors (PIs) and one associated with NNRTIs. Mutations associated with resistance to integrase strand transfer inhibitors (INSTIs) were not detected in this study (Table 5).

**TABLE 5:** Drug resistance mutations detected by Sanger sequencing method

Patient	HIV subtype	Mutation	Region^*a*^	Result using indicated protocol		Drug resistance profile^*b*^
				Standard	CODEHOP	
1	CRF01_AE	E138A	RT	+	+	LLR (RPV), PLLR (ETR), HLR (FTC, 3TC), LLR (ABC)
		M184V	RT	+	+	
2	CRF01_AE	K70E	RT	+	+	LLR (TDF), HLR (FTC, 3TC), IR (ABC)
		M184V	RT	+	+	
3	C	M184V	RT	+	+	HLR (FTC, 3TC), LLR (ABC)
4	B	L90M	PR	+	+	LLR (ATV/r, LPV/r), HLR (EFV, NVP)
		K103N	RT	+	+	
5	CRF43_02G	V179E	RT	+	+	PLLR (EFV, ETR, NVP, RPV)

a RT, reverse transcriptase; PR, protease.

b LLR, low-level resistance; HLR, high-level resistance; IR, intermediate resistance; PLLR, potential low-level resistance; RPV, rilpivirine; ETR, etravirine; FTC, emtricitabine; 3TC, lamivudine; ABC, abacavir; TDF, tenofovir; ATV/r, atazanavir/ritonavir; LPV/r; lopinavir/ritonavir; EFV, efavirenz; NVP, nevirapine.

### Detection of minority variants by MinION sequencing.

The sensitivity of CODEHOP-mediated PCR to detect minority variants at protease and reverse transcriptase positions associated with drug resistance was compared to that of the standard RT-PCR protocol using MinION sequencing (Oxford Nanopore Technology), as described in Materials and Methods. The overall mean read depth of genome coverage was significantly higher with the CODEHOP scheme than with the standard scheme (*P = *0.009), resulting in higher genome coverage, at 100-fold (Table 6). In either scheme, there was no significant difference in the percentages of mapped reads and depths of coverage between different HIV-1 genotypes (data not shown). All DRMs detected by the Sanger method were also detected by MinION sequencing. The analysis of minority variants showed additional DRMs detected in 4 (11.4%) treatment-experienced patients (patients 1, 2, 6, and 7) using the CODEHOP scheme and in only 1 (2.8%) treatment-experienced patient (patient 2) using the standard scheme, resulting in a 97.5% overall agreement between the two schemes, with a kappa value of 0.84 (95% CI, 0.59 to 0.95; *P < *0.001) (Table 7). The overall prevalences of DRMs were 17.1% in treatment-experienced patients and 1.2% in treatment-naive patients. The minority DRMs were detected at read count frequencies ranging from 31.7% to 47.8%, and the depths of coverage varied between 41- and 222-fold. Overall, reads with DRMs were detected at a higher frequency with the CODEHOP scheme (74.8%) than with the standard scheme (58.8%; *P = *0.014). Among the minority variants, V106I (a change of V to I at position 106) and E138G are usually associated with low-level resistance to NNRTIs, D67N confers low-level resistance to the NRTI zidovudine, and M46L may reduce susceptibility to some PIs. All patients but one with DRMs in the reverse transcriptase region had no viral suppression with a history of treatment with reverse transcriptase inhibitors (Table 8).

**TABLE 6:** Comparison of HIV-1 *pol* gene MinION sequencing between standard and CODEHOP schemes after barcode demultiplexing

Scheme	Avg result (range) for:				
	Total no. of reads	No. of mapped reads	% of mapped reads	% coverage (>100×)	Read depth (range)
Standard	7,197 (5,014–10,339)	5,503 (3,933–8,520)	76.1 (59.8–86.1)	75.6 (75.3–76.1)	1,556 (914–2,419)
CODEHOP	6,206 (4,217–9,914)	4,920 (1,819–8,684)	78.9 (43.1–92.1)	89.4 (89.1–89.5)	1,928 (703–3,423)

**TABLE 7:** Consensus and minority variants associated with antiretroviral drug resistance

Patient	HIV subtype	Mutation	Region^*a*^	Result using:							
				Sanger sequencing		Oxford Nanopore Technology^*b*^					
				Standard	CODEHOP	Standard			CODEHOP		
						F (%)	DP	Q	F (%)	DP	Q
1	CRF01_AE	V106I	RT	−	−	—	—	—	37.5	127	30
		E138A	RT	+	+	95.7	578	61	96.5	1,058	61
		M184V	RT	+	+	95.1	506	58	94.1	1,075	48
2	CRF01_AE	D67N	RT	−	−	37.8	125	28	47.8	222	31
		K70E	RT	+	+	93.3	541	51	95	1,013	57
		M184V	RT	+	+	77	886	23	92.7	1,107	42
3	C	M184V	RT	+	+	50.2	480	41	87.3	822	39
4	B	L90M	PR	+	+	99	882	57	98	829	59
		K103N	RT	+	+	99	1,051	63	99	936	63
5	CRF43_02G	V179E	RT	+	+	NA	N/A	NA	100	712	53
6	CRF01_AE	M46L	PR	−	−	—	—	—	43.2	95	27
7	CRF01_AE	E138G	RT	−	−	—	—	—	31.7	41	25

a RT, reverse transcriptase; PR, protease.

b F, variant frequency; DP, depth of coverage; Q, per-base sequencing quality score as determined by Nanopolish tool; —, not detected; NA, not available.

**TABLE 8:** Treatment histories of patients with drug resistance mutations

Patient	Age (yr)	Sex^*a*^	HIV-1 subtype	Viral load (copies/ml)	DRM(s)^*b*^	Resistance to^*c*^:	Treatment^*c*^	
							Current	Previous
1	44	M	CRF01_AE	440,786	V106I, E138A, M184V	NNRTIs, NRTIs	1 INSTI + 2 NRTIs	1 NNRTI + 2 NRTIs
2	40	M	CRF01_AE	13,959	D67N, K70E, M184V	NRTIs	1 INSTI + 2 NRTIs	1 NNRTI + 2 NRTIs
3	9	M	C	1,099	M184V	NRTIs	1 INSTI + 2 NRTIs	2 NRTIs
4	26	M	B	13,319	L90M, K103N	PIs, NNRTIs	None	None
5	50	M	CRF43_02G	159,523	V179E	NNRTIs	1 INSTI + 2 NRTIs	1 NNRTI + 2 NRTIs
6	38	M	CRF01_AE	47,867	M46L	PIs	1 INSTI + 2 NRTIs	1 NNRTI + 2 NRTIs
7	31	M	CRF01_AE	170,227	E138G	NNRTIs	1 INSTI + 2 NRTIs	1 NNRTI + 2 NRTIs

a M, male.

b DRM, drug resistance mutation.

c INSTI, integrase strand transfer inhibitor; NNRTIs, nonnucleoside analogue reverse transcriptase inhibitors; NRTIs, nucleoside/nucleotide-analogue reverse transcriptase inhibitors; PIs, protease inhibitors.

## DISCUSSION

The j-CODEHOP tool was used in this study to design universal primers that targeted the conserved regions of the *pol* gene of different HIV-1 subtypes and CRFs. While previous studies tested newly designed primers on only major subtypes, CODEHOPs were validated on 18 different subtypes and CRFs that represent diverse geographical regions around the world (2). A clear amplification product could be obtained using a single pair of primers in each PCR step for all HIV-1 isolates tested. Multiplexed PCR with pooled primers, a classical approach with the CODEHOP protocol (17), generated no satisfactory results. This is somewhat expected due to the high number of primers in the pool, with an increased possibility of primer dimer formation and nonspecific primer annealing. Using amplification kits designed specifically for endpoint multiplex PCR that are available in the market would overcome this problem and generate better results, but at higher cost. Furthermore, considering the high failure rate of the standard protocol and the potential requirement of a two-step nested PCR, the turnaround time and cost of the CODEHOP protocol were significantly reduced compared to those of the standard protocol.

Using the standard amplification scheme, consensus variants were detected at frequencies ranging from 50% to 99%, while this range was significantly higher (87% to 100%) with the CODEHOP-mediated genotyping. Minority variants were detected at frequencies higher than 30% with the two amplification schemes. All nucleotide variants were indeed assessed at a frequency cutoff of 20%, as below this read count frequency, variant calling is not reliable due to the high error rate of nanopore sequencing (20). PCR error can also account for the misinterpretation of single-nucleotide variants; however, at a 20% frequency threshold and using PCR enzymes with proofreading activity, this assumption becomes less plausible.

Since only variants resulting in nonsynonymous amino acid changes at drug resistance positions were considered for this study, the number of minority variants reported was too low to compare the sensitivities of the two amplification schemes in the detection of minority DRMs. However, since the read depth of genome coverage was significantly higher with the CODEHOP scheme, the sensitivity of CODEHOP-mediated PCR for detecting minority variants is expected to exceed that of the standard PCR protocol. Primer replacement has indeed been shown to improve the genome coverage and sensitivity of next-generation sequencing (21).

The concordance of drug resistance profiles between Sanger-based sequencing and MinION sequencing was 100% in treatment-naive patients and 88.6% in treatment-experienced patients. Despite the detection of minority DRMs in treatment-experienced patients at read frequencies higher than 30% by MinION sequencing, they were not detected by Sanger-based sequencing. The minority variants described in this study were not located in homopolymer regions generally associated with false base calling on different NGS platforms, including the Oxford Nanopore MinION (22). In addition, they were supported by a depth of coverage at drug resistance sites of >40, per-base sequencing quality of >20, and read frequency of >30%. One possible explanation of this discordance is the bias in the selection of variants by the sequencing primers used during the sequencing reactions in the Sanger method. In addition, Sanger-based sequencing generally fails to detect variants at frequencies lower than 20% (23). However, this cutoff is based on comparative studies investigating mutations in human tumor samples where highly concentrated DNA templates were used, and, therefore, we cannot exclude the potential failure of Sanger-based sequencing to detect DRMs in heterogeneous viral populations at frequencies above that threshold. Indeed, only 76% of the majority of HIV-1 variants at a cutoff of ≥20% could be detected by the Sanger method, as previously reported (8). Moreover, our results partly corroborate previous findings, underlining the inferiority of Sanger sequencing in detecting majority and minority DRMs compared to their detection by NGS (8, 9, 24).

One concern with the detection of minority variants by MinION sequencing is the high frequency threshold used to consider data reliable. The sensitivity of detection of the minority variants would be reduced further with a lower plasma viral load or when multiplexing a high number of samples. Increasing the sample DNA concentration in the sequencing library, reducing the number of samples per run, and increasing the depth of genome coverage via increasing the sequencing time would improve sensitivity.

CODEHOP-mediated PCR was able to amplify and genotype HIV-1 isolates from clinical plasma samples with a viral load as low as 272 copies/ml. The analytical sensitivity of the technique was ∼137 copies/ml for the protease/reverse transcriptase gene and ∼117 copies/ml for the integrase gene. This sensitivity is higher than those of the current FDA-approved genotyping techniques, like the Sentosa SQ HIV genotyping assay (Vela Diagnostics, USA), which uses next-generation-sequencing technology with a limit of detection of 1,000 copies/ml, and the ViroSeq HIV-1 genotyping system (Abbott Laboratories, USA), based on Sanger sequencing and using plasma viral loads ranging from 2,000 to 750,000 copies/ml. We believe, however, that the limit of detection varies in part with the HIV-1 subtype, depending on the number of mismatches between the primers and the viral genome.

CODEHOPs performed better than the standard degenerate primers in one-step nested PCR, with success rates of up to 98% versus 84%, respectively, despite using the same PCR kits and the same RNA template. By performing a two-step RT-PCR followed by a nested/seminested PCR, the CODEHOP method yielded a 100% PCR success rate. The superiority of the two-step RT-PCR over the one-step can be explained by an enhanced primer binding efficiency due to the reduction of secondary structures mediated by the introduction of a preheating step and by the presence of 1% formamide (25–27). Previous studies have reported success rates of HIV-1 genotyping ranging from 60% to 97% (1, 28, 29). The main challenge to successful genotyping is a plasma viral load of <1,000 copies/ml. However, when there is enough RNA template, low primer binding efficiency, poor sample quality, technical artifacts, or poor performance of the PCR assays are potential causes of PCR failure, as previously suggested (1, 30).

DRMs were found in ∼17% of treatment-experienced patients. These DRMs were correlated with virologic failure, and their associated drug resistance profiles with the patient’s treatment history. For instance, patient 1, who had one NRTI DRM and two NNRTI DRMs, had evidence of exposure to both NRTIs and NNRTIs. Interestingly, patient 7, with a history of treatment with an NNRTI-based regimen, had only a low-frequency E138G mutation that is usually associated with low-level resistance to NNRTIs. Several studies have evaluated the effects of low-frequency DRMs on the rates of antiretroviral treatment failure. NNRTI-resistant minority variants have been shown to be associated with an increased risk of virologic and treatment failure. The higher the DRM frequency, the higher was the risk of virologic failure (31). The detection of minority NNRTI-associated mutations in treatment-naive patients has also been shown to increase two to three times the risk of virologic failure after starting an NNRTI-based regimen (32).

Low adherence to antiretroviral treatment is generally associated with an increased risk of virologic failure (33). In particular, when controlled for the presence of minority DRMs, less than 60% adherence to NNRTIs was associated with a 1.7-times increase in the risk of virologic failure (34). Among our cohort, only patient 2 had a history of noncompliance with treatment. In addition, he had three NRTI-associated mutations, including one, D67N, detected only by MinION sequencing, at ∼48% frequency. This additional mutation had no significant impact on the predicted drug susceptibility, since D67N, associated with low-level resistance to zidovudine (AZT), occurred simultaneously with two other DRMs, K70E and M184V, that increase susceptibility to AZT (35). A previous study reported substantial changes in the predicted susceptibility to NRTIs and NNRTIs in 55.5% of patients following the detection of DRMs by next-generation sequencing (36).

Of note, M46L, a mutation potentially associated with low-level resistance to some PIs, was detected at ∼43% frequency in a patient (patient 6) who had not been exposed to PIs. M46L has been reported as a minority variant detected in treatment-naive (37) and -experienced (38) patients. Its contribution to the virological breakthrough following treatment with PIs is unknown. However, minority variants in the protease region have not been shown to increase the risk of virologic failure or to affect the efficacy of first-line treatment with PIs (39), possibly due to the high genetic barrier to resistance that is conferred by boosted PIs (40).

### Conclusions.

Primers designed using the CODEHOP approach show promise as an alternative to the standard degenerate and nondegenerate primers for HIV-1 genotyping using either Sanger-based or next-generation sequencing. Variants missed by Sanger-based and MinION sequencing using the standard primers were found at frequencies higher than 30% and were mostly correlated with the treatment regimens. However, more studies with more samples are needed to assess the clinical relevance of minority variants detected by CODEHOP-mediated PCR and missed by classical PCR methods.

## MATERIALS AND METHODS

### Ethics statement.

The research study was carried out in accordance with the recommendations of the Ethical Decision Committee of the Research Administration, Faculty of Medicine, Kuwait University, and the 2008 Declaration of Helsinki. An informed consent was obtained from each patient before blood sample collection.

### Standard HIV-1 genotyping.

Total RNA was isolated from plasma of patients with HIV-1 infection using the MagNa pure LC 2.0 system (Roche Diagnostic Systems, Branchburg, NJ, USA). Plasma viral load was determined using the COBAS AmpliPrep/COBAS TaqMan HIV-1 test, version 2.0 (Roche Diagnostic Systems). Amplification of the HIV-1 *pol* gene was performed by nested RT-PCR using previously described primers (41, 42). Protease and reverse transcriptase genes were amplified according to the World Health Organization (WHO) recommended protocol (41), in two overlapping amplicons with amplicon sizes of 836 bp and 776 bp, whereas the expected amplicon size for the integrase gene was 1,253 bp. The standard amplification protocol included one-step RT-PCR using the SuperScript III one-step RT-PCR system with Platinum *Taq* DNA polymerase (Invitrogen, Life Technologies, Van Allen Way, Carlsbad, CA), followed by a nested PCR using the *Taq* PCR master mix (Qiagen, Hilden Germany). The first RT-PCR was performed using a gradient PCR approach that included 45 min of reverse transcription at 37°C, 2 min of reverse transcriptase inactivation and initial denaturation at 98°C, and 35 cycles consisting of 1 min of denaturation at 95°C, 1 min of annealing at 35 to 45°C, and 2 min of extension at 68°C, followed by 10 min of final extension at 68°C. The cycling parameters for the second PCR included 5 min of initial denaturation at 95°C and 35 cycles consisting of 1 min of denaturation at 95°C, 1 min of annealing at 55°C, and 2 min of extension at 68°C, followed by 10 min of final extension at 68°C. When one-step RT-PCR failed, a two-step nested RT-PCR was carried out using the high-capacity cDNA reverse transcription kit (Applied Biosystems, Foster City, CA, USA) and the *Taq* PCR master mix kit (Qiagen) in the presence of 1% Hi-Di formamide (Applied Biosystems) (25). PCR products were visualized by gel electrophoresis on 1% agarose gels using a UV transilluminator (UVP [Ultra-Violet Products Ltd.], Cambridge, UK). Sanger-based sequencing was then carried out by direct double-strand DNA cycle sequencing of the purified amplicons using the ABI Prism BigDye terminator cycle sequencing kit, version 3.1 (Applied Biosystems), on the ABI 3500 genetic analyzer (Applied Biosystems). Mutations associated with resistance to protease, reverse transcriptase, and integrase inhibitors were identified using the Stanford University genotypic resistance interpretation algorithm (35, 43).

### CODEHOP-mediated RT-PCR.

The amino acid sequences of the *pol* gene from 13 HIV-1 M group subtypes (A1, A2, A3, A4, B, C, D, F1, F2, G, H, J, and K) and 96 circulating recombinant forms (CRFs) were obtained from NCBI GenBank and the Los Alamos National Laboratory HIV Sequence Database (3) and loaded into MEGA X (44). CODEHOPs were designed to amplify the protease, reverse transcriptase, and integrase regions in nested or seminested RT-PCR, using the j-CODEHOP tool as described earlier (14, 16, 17). To reduce the overall degeneracy of degenerate primer sets, dITP (I) was used to substitute for any N (A, C, G, or T) (45). CODEHOPs were either pooled and tested in multiplexed reactions or used in single-primer-pair reactions in an attempt to detect 18 different available HIV-1 subtypes (A, B, C, G, CRF01_AE, CRF02_AG, CRF06_cpx, CRF07_BC, CRF08_BC, CRF10_CD, CRF16_A2D, CRF25_cpx, CRF32_06A1, CRF35_AD, CRF43_02G, CRF50_A1D, CRF63_02A1, and CRF67_01B) that had been previously identified in the clinical samples using the standard protocol. Only plasma samples with viral loads ranging between 10^3^ and 10^5^ copies/ml were selected for the evaluation step. All primers were purchased from Thermo Fisher Scientific (Waltham, MA, USA). The HIV-1 *pol* gene was amplified with one- or two-step nested/seminested RT-PCR using the same conditions described above. Sanger-based sequencing was then carried out to confirm the specificity of the amplified products as described above.

### Validation of the CODEHOP-mediated PCR in HIV-1 genotyping.

The validation panel used to calculate the sensitivity and specificity of CODEHOP-mediated RT-PCR consisted of 100 randomly selected samples that were routinely tested positive by the standard HIV-1 genotyping protocol and 100 samples from HIV-1-seronegative patients. A head-to-head evaluation of the CODEHOP protocol and the standard protocol was also performed on 121 samples prospectively collected from treatment-naive (*n* = 86) and treatment-experienced patients (*n* = 35) with HIV-1 infection. The analytical sensitivity of the assay, also known as the limit of detection (LOD), was determined by probit regression analysis using 12 replicates of each dilution of the HIV-1 standard (ATCC VR-3245SD; ATCC, Manassas, VA, USA). The analytical specificity was determined using clinical samples positive for human T-cell lymphotropic virus type 1 (HTLV-I) RNA, hepatitis B virus DNA, or hepatitis C virus RNA and a panel of viruses obtained from ATCC, including herpes simplex virus types 1 and 2, human herpesvirus 6, cytomegalovirus, varicella zoster virus, Epstein-Barr virus, rubella virus, coxsackievirus B4, influenza A and B viruses, parainfluenza virus 1, adenovirus C5, rhinovirus B14, respiratory syncytial virus, and coronavirus OC43.

### Detection of minority variants by MinION sequencing.

The detection sensitivity of minority variants by the CODEHOP protocol was compared to that of the standard protocol using nanopore next-generation sequencing. Total RNAs isolated from plasma samples of patients were subjected to nested or seminested RT-PCR to amplify the *pol* gene, using the SuperScript III one-step RT-PCR system with Platinum *Taq* high-fidelity DNA polymerase (Invitrogen) for the one-step RT-PCR and Platinum SuperFi PCR master mix (Invitrogen) for the nested/seminested PCR. The DNA concentrations in the PCR products were measured using the Qubit double-stranded DNA (dsDNA) high-sensitivity assay (Thermo Fischer Scientific). A total of 11 samples (100 ng/sample) plus one no-template negative control were multiplexed per sequencing run using the rapid barcoding sequencing kit (Oxford Nanopore Technologies, Oxford, UK). The library was loaded onto an R9.4.1 flow cell, and the sequencing was performed on a MinION MK1b instrument using MinKNOW version 21.02.1 (Oxford Nanopore Technologies). The sequencing run was monitored using RAMPART version 1.2.0 (46) and stopped when at least ∼300-fold genome coverage was achieved across all samples. Guppy Base Caller Software version 4.5.2 (Oxford Nanopore Technologies) with high accuracy mode enabled was then used for base calling, demultiplexing, and barcode trimming of fast5 reads that attained the minimum quality threshold. The quality of fastq reads was checked with FastQC (47) and Nanoplot (48). Reads shorter than 100 bases were filtered out. To determine the consensus sequence in each sample, the Medaka workflow (49) described in the ARTIC Network Bioinformatics pipeline (20) was followed using the HIV-1 HXB2 strain (accession number K03455) as the reference. The proportion of mapped reads and the average depth of genome coverage were assessed for each sample using SAMtools (50). Variant calling and annotation of variant call format (VCF) files were performed to determine the quality and the frequency of consensus variants. The consensus sequence was then used to align fastq reads using minimap2 (51) before calling minority variants with Nanopolish (52) and Varscan2 (53), assuming a depth threshold of 20-fold and 20% minimum variant frequency. Adjacent nucleotide variants (multinucleotide polymorphism [MNP]) within a single codon were identified using the HaplotypeCaller algorithm in the Genome Analysis Toolkit (GATK) version 4.2.0 (54) and were annotated with SnpSift (55). To confirm that they coexisted in the same viral quasispecies, variant phasing and read haplotyping were carried out for all samples with MNP at a drug resistance site using the WhatsHap tool (56). Visualization of the phasing results was performed on the Integrative Genomics Viewer (IGV) version 2.10.0 (57). Variant calls in the VCF files were used to create the final consensus sequence using *bcftools consensus* (58). HIV-1 sequences from patients with variants associated with antiretroviral drug resistance were available at GenBank under the accession numbers OK070793 to OK070799.

### Statistical analysis.

The chi-square test or Fischer’s exact test was used to compare the proportions of PCR failure rates between the CODEHOP method and the standard method. Probit regression analysis was used to determine the limit of detection of the CODEHOP-mediated PCR in HIV-1 genotyping. The two-tailed paired *t* test was used to compare the continuous data between the CODEHOP method and the standard method in MinION sequencing. A test result with a *P* value of <0.05 was considered statistically significant. All the statistical analyses were performed using IBM SPSS Statistics for Windows, version 25 (IBM Corp., Armonk, NY, USA).
