# Supplementary material for: CODEHOP-Mediated PCR Improves HIV-1 Genotyping and Detection of Variants by MinION Sequencing
Source: Microbiol Spectr. 2021 Oct 20;9(2):e01432-21. doi: 10.1128/Spectrum.01432-21 (PMC8528109; doi:10.1128/Spectrum.01432-21)
Supplement: SUPPLEMENTAL FILE 1 — Supplemental material. Download SPECTRUM01432-21_Supp_1_seq1.pdf, PDF file, 0.1 MB [file spectrum01432-21_supp_1_seq1.pdf]

Supplementary Material

Table S1. HIV-1 primers designed using j-CODEHOP tool

Target	Sense	Position*	Sequence (5’→3’)
Protease	Forward	1860-1879	CCATAAAGCAAGRGTKTTRG
	Reverse	2804-2784	CTGAGTTCTCTTRTTRARYTC
	Forward	2013-2032	TAGGAAAAARGGYTGTTGGA
	Reverse	2774-2754	TACTAATTTTCTCCAYTTIGT
	Reverse	2738-2718	TATGGCAAAYAYTGGIGTRTT
	Forward	1857-1876	CGGCCATAARGCAAGRGTKT
	Reverse	2759-2739	TTTAGTACTGTCYTTYTTYTT
	Forward	1866-1885	GGCAAGAGTKTTGGCTGARG
	Reverse	2651-2631	TAATGCTTTTATYTTYTCTTC
	Forward	2136-2153	TCAGAGCAGRCYRGARCC
	Reverse	2627-2607	CAATGGCCATTGYTTIACYTT
Reverse Transcriptase	Forward	2319-2338	CTATTAGAYACAGGRGCIGA
	Reverse	3772-3754	GCTTGCCARTAITCYVICC
	Forward	2418-2436	GTAAGACARTATGAIVAIR
	Reverse	3707-3687	AGTCTTTCCCCADATIACDAT
	Forward	2469-2488	GGTACAGTRTTRGTIGGICC
	Reverse	3673-3655	ATTTTTTGIMCIRCITCTG
	Forward	2415-2434	AAAGTAAGACARRATGAKVA
	Reverse	3743-3723	CCATGTTTCTTTYGKKATRGG
	Forward	2430-2449	GATCAGATACYYRTAGAVAT
	Reverse	3716-3696	AAATTTAGGRRTYTTYCCCCA
	Forward	2478-2497	TTAGTAGGACCTACVCCTRT
	Forward	2739-2759	AAGAAAAAAGAYAGYACYAAR
Integrase	Forward	4062-4081	ATCATTCAAGCACARCCIGA
	Reverse	5666-5649	CCTAGGAAARTGICKIAC
	Forward	4152-4174	TGGGTACCAGCACAYAARGGIAT
	Reverse	5597-5580	TTCCCTCTGIGGICCYTG
	Forward	4165-4186	ACAAAGGIATTGGIGGIAAYGA
	Reverse	5576-5559	TTCTGGGGCYTGYTCCAT
	Forward	4056-4075	TTAGGAATCATTCARGCACA
	Reverse	5446-5430	GATATTCACAMCTABGD
	Forward	4077-4096	CCAGATAAGAGTGARKCAGA
	Reverse	5393-5373	GATTCTGAAAAACARTYAAAR
	Forward	4158-4177	CCAGCACAYAARGGRATTGG
	Reverse	5213-5193	GGGATGTGTACTTCTGARCTT

* Primer position according to the HIV-1 HXB2 reference strain (accession no. K03455)

Table S2. Validation of CODEHOP-mediated PCR in HIV-1 genotyping

No	Year	HIV-1 subtype	Viral load (copies/ml)	No	Year	HIV-1 subtype	Viral load (copies/ml)
1	2020	CRF06_cpx	795094	51	2019	CRF01_AE	112000
2	2020	B	20949	52	2019	CRF01_AE	369000
3	2020	A	100969	53	2019	A	2630
4	2021	B	15479	54	2019	B	18173
5	2021	CRF02_AG	12168	55	2019	C	3959
6	2020	CRF01_AE	N/A*	56	2019	C	4441
7	2020	C	N/A	57	2020	C	1879
8	2021	CRF02_AG	55901	58	2019	CRF02_AG	265000
9	2021	CRF02_AG	17484	59	2019	C	1099
10	2021	CRF02_AG	151847	60	2019	CRF06_cpx	156000
11	2021	CRF02_AG	614643	61	2019	C	18900
12	2020	G	N/A	62	2019	CRF06_cpx	592000
13	2020	C	N/A	63	2020	CRF02_AG	15900
14	2020	A	N/A	64	2020	CRF43_02G	15753
15	2020	CRF01_AE	N/A	65	2020	CRF02_AG	14549
16	2020	CRF01_AE	Negative	66	2019	C	1750000
17	2019	CRF02_AG	N/A	67	2020	CRF01_AE	1100000
18	2020	B	N/A	68	2019	C	33415
19	2020	C	2328	69	2019	C	80655
20	2020	CRF06_cpx	18289	70	2019	CRF02_AG	61579
21	2020	C	18289	71	2019	C	141995
22	2020	CRF01_AE	29266	72	2019	B	20467
23	2020	CRF01_AE	172305	73	2019	CRF02_AG	8020
24	2020	CRF01_AE	N/A	74	2019	CRF01_AE	49200
25	2020	CRF16_A2D	5860	75	2020	B	13319
26	2020	CRF06_cpx	555000	76	2020	CRF01_AE	27079
27	2019	CRF01_AE	95621	77	2020	CRF07_BC	2400
28	2019	CRF02_AG	1959598	78	2020	CRF07_BC	58336
29	2020	CRF02_AG	79100	79	2020	CRF02_AG	5777
30	2020	C	N/A	80	2020	CRF02_AG	N/A
31	2020	CRF02_AG	24257	81	2020	CRF01_AE	1960
32	2020	CRF02_AG	272	82	2020	CRF06_cpx	181622
33	2019	B	63939	83	2020	B	41432
34	2020	B	N/A	84	2020	CRF07_BC	4390
35	2020	B	49900	85	2020	CRF02_AG	47028
36	2020	C	95734	86	2020	CRF02_AG	24429
37	2020	C	16914	87	2020	B	10489
38	2020	B	120732	88	2020	CRF01_AE	240932
39	2020	CRF02_AG	17350	89	2019	CRF35_AD	1019
40	2020	CRF01_AE	152731	90	2020	CRF25_cpx	1184476
41	2020	C	124499	91	2018	CRF63-02A1	72060
42	2020	CRF01_AE	N/A	92	2018	CRF50-A1D	521684
43	2020	B	90500	93	2019	CRF08_BC	13116

No	Year	HIV-1 subtype	Viral load (copies/ml)	No	Year	HIV-1 subtype	Viral load (copies/ml)
44	2019	B	N/A	94	2019	CRF07_BC	213000
45	2019	CRF01_AE	3194	95	2020	B	N/A
46	2019	C	87259	96	2019	CRF16_A2D	32982
47	2021	CRF02_AG	61196	97	2018	CRF43-02G	195523
48	2020	CRF02_AG	20526	98	2019	CRF10_CD	200577
49	2020	CRF07_BC	19199	99	2019	CRF02_AG	378346
50	2020	CRF02_AG	7706	100	2020	C	331757

*N/A, not available

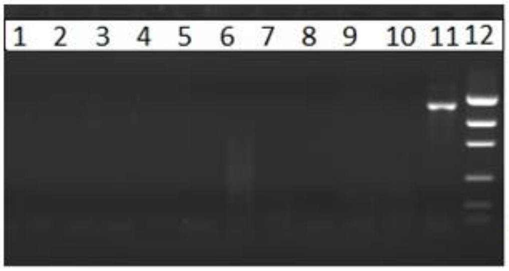

Figure S1. Analytical specificity of CODEHOP-mediated PCR amplification of protease/reverse transcriptase regions. Lane 1: human T- lymphotropic virus-1; lane 2: hepatitis B virus; lane 3: hepatitis C virus; lane 4: Epstein-Barr virus; lane 5: herpes simplex virus 1; lane 6: herpes simplex virus 2; lane 7: cytomegalovirus; lane 8: varicella-zoster virus; lane 9: coxsackievirus B4; lane 10: negative control; lane 11: HIV-1 positive control; lane 12: high DNA mass ladder.
